# The effects of exercise, heat-induced hypo-hydration and rehydration on blood–brain-barrier permeability, corticospinal and peripheral excitability

**DOI:** 10.1007/s00421-024-05616-x

**Published:** 2024-09-28

**Authors:** Nasir Uddin, Jamie Scott, Jonathan Nixon, Stephen D. Patterson, Dawson Kidgell, Alan J. Pearce, Mark Waldron, Jamie Tallent

**Affiliations:** 1 Faculty of Sport, Technology and Health Sciences, St Mary’s University, Twickenham, UK; 2 School of Sport, Rehabilitation, and Exercise Sciences, University of Essex, Wivenhoe Park, Colchester, CO4 3SQ UK; 3 Nuffield Department of Clinical Neurosciences, University of Oxford, Oxford, UK; 4 Monash Exercise Neuroplasticity Research Unit, Department of Physiotherapy, School of Primary and Allied Health Care, Faculty of Medicine, Nursing and Health Science, Monash University, Melbourne, Australia; 5 Swinburne Neuroimaging Facility, School of Health Science, Swinburne University of Technology, Melbourne, Australia; 6 Applied Sport, Technology, Exercise and Medicine, College of Engineering, Swansea University, Swansea, Wales UK; 7 School of Health and Behavioural Sciences, University of the Sunshine Coast, Sippy Downs, QLD Australia; 8 Welsh Institute of Performance Science, Swansea University, Swansea, UK

**Keywords:** Dehydration, Neuromuscular function, Thermoregulation, Transcranial magnetic stimulation, Rapid weight loss

## Abstract

**Purpose:**

The effects of low-intensity exercise, heat-induced hypo-hydration and rehydration on maximal strength and the underlying neurophysiological mechanisms are not well understood.

**Methods:**

To assess this, 12 participants took part in a randomised crossover study, in a prolonged (3 h) submaximal (60 W) cycling protocol under 3 conditions: (i) in 45 °C (achieving ~ 5% body mass reduction), with post-exercise rehydration in 2 h (RHY2), (ii) with rehydration across 24 h (RHY24), and (iii) a euhydrated trial in 25 °C (CON). Dependent variables included maximal voluntary contractions (MVC), maximum motor unit potential (M_MAX_), motor evoked potential (MEP_RAW_) amplitude and cortical silent period (cSP) duration. Blood–brain-barrier integrity was also assessed by serum Ubiquitin Carboxyl-terminal Hydrolase (UCH-L1) concentrations. All measures were obtained immediately pre, post, post 2 h and 24 h.

**Results:**

During both dehydration trials, MVC (RHY2: *p* < 0.001, RHY24: *p* = 0.001) and MEP_RAW_ (RHY2: *p* = 0.025, RHY24: *p* = 0.045) decreased from pre- to post-exercise. MEP_RAW_ returned to baseline during RHY2 and CON, but not RHY24 (*p* = 0.020). MEP/M_MAX_ ratio decreased across time for all trials (*p* = 0.009) and returned to baseline, except RHY24 (*p* < 0.026). Increased cSP (*p* = 0.011) was observed during CON post-exercise, but not during RHY2 and RHY24. Serum UCH-L1 increased across time for all conditions (*p* < 0.001) but was not significantly different between conditions.

**Conclusion:**

Our findings demonstrate an increase in corticospinal inhibition after exercise with fluid ingestion, but a decrease in corticospinal excitability after heat-induced hypo-hydration. In addition, low-intensity exercise increases peripheral markers of blood–brain-barrier permeability.

**Graphical abstract:**

**Figure:**
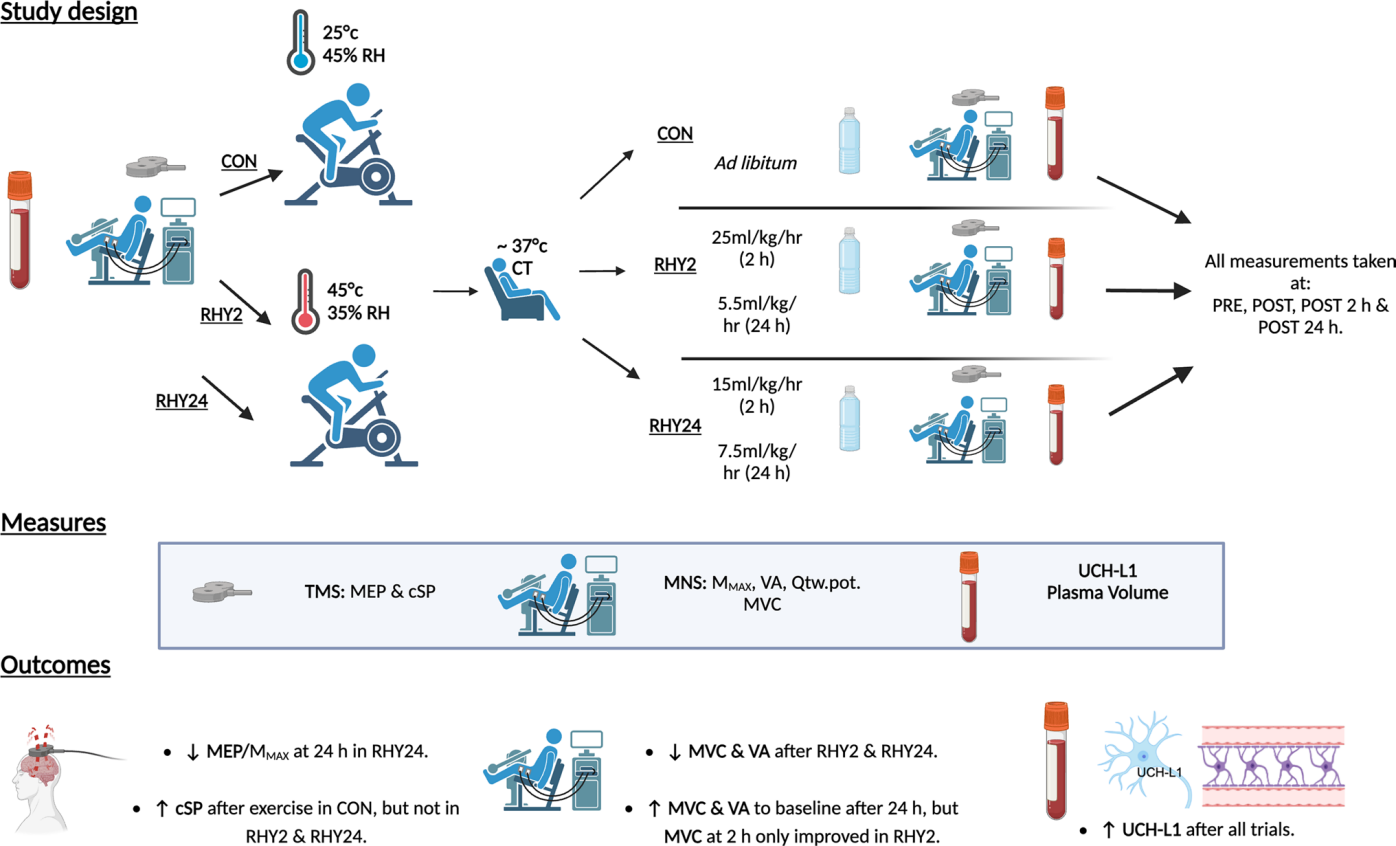
The mechanisms and time-course of change in neuromuscular function after intracellular dehydration and subsequent rehydration, are not well understood. In this present study, twelve healthy participants underwent a control trial (CON) and two experimental trials in heat (45 °C, 45% relative humidity [RH]) to achieve a 5% reduction in body mass via dehydration and low-intensity cycling (60 W), then rehydrated rapidly (RHY2) or progressively (RHY24). Participants underwent various measures of transcranial magnetic stimulation (TMS) and peripheral motor nerve stimulation (MNS) before (PRE), after core temperature (CT) returned to baseline (POST), and after 2 h and 24 h. These measures included: motor evoked potential amplitude (MEP), TMS-evoked cortical silent period (cSP), compound muscle action potential (M_MAX_), voluntary activation (VA), potentiated twitch (Qtw,pot), maximal voluntary contractions (MVC) and serum Ubiquitin carboxyl-terminal hydrolase (UCH-L1). The novel finding was a different corticospinal response to low-intensity exercise (i.e., higher corticospinal inhibition when hydrated) and rehydration strategies (i.e., lower corticospinal excitability after gradual rehydration).

**Supplementary Information:**

The online version contains supplementary material available at 10.1007/s00421-024-05616-x.

## Introduction

Water plays a crucial role in cellular homeostasis, with transient losses of dissolved substances in body fluid leading to alterations in osmolality and, consequently, water distribution across neural and skeletal muscle cell membranes (Hargreaves and Febbraio 1998; Verbalis 2010). Typical thermoregulatory sweating, coupled with inadequate fluid intake, can result in hypotonic fluid losses from extracellular fluid in relation to blood plasma. It has long been established that this leads to an osmotic gradient, facilitating transmembrane flow of fluid from the intracellular fluid space towards the extracellular fluid space (Costill et al. 1976; Durkot et al. 1986). This process of intracellular fluid loss (and the hypertonic characteristics of the extracellular fluid) is referred to as intracellular dehydration, hypertonic hypovolemia (Adolph et al. 1947; Lee and Mulder 1935; Pearcy et al. 1956) and has likely implications on the central (CNS) and peripheral nervous systems (PNS), which might result in decreased muscle function, but a definitive in-vivo mechanism remains unknown (Cheuvront and Kenefick 2014).

Hypo-hydration notably reduces exercise performance via increased cardiovascular strain (González-Alonso et al. 1997), reduced blood flow, aerobic metabolism (Cheuvront et al. 2010) and thermoregulatory function (Casa 1999). However, the neuromuscular (NM) responses to hypo-hydration are less well understood and evidence for a loss of maximal strength is mixed (for review, see Cheuvront and Kenefick 2014). This could be due to limitations and inconsistencies in the methodology of various dehydration studies. Such limitations include accounting for fluid restoration (Cheuvront et al. 2006; Bowtell et al. 2013), type of dehydration e.g., active *vs.* passive or fluid restriction *vs.* fluid loss (Pallares et al. 2016; Segikuchi et al. 2022), and a vast variation in measures of NM function (Bigard et al. 2001; Evetovich et al. 2002; Bowtell et al. 2013; Barleyet al. 2018a).

Relatively few studies have used transcranial magnetic stimulation (TMS) and peripheral motor nerve stimulation (MNS) techniques to provide mechanistic reasoning for the reduction (or lack of change) in maximal strength after heat-induced hypo-hydration (for review, see Cheuvront and Kenefick 2014). While it has been reported that Voluntary Activation (VA) is unaffected (Del Coso et al. 2008; Periard et al. 2012; Barley et al. 2018a), Bowtell et al. (2013) speculated that hypo-hydration (3% body mass reduction) resulted in different neural strategies (relative to the euhydrated group) to preserve motor output, as observed through unchanged corticospinal inhibition and increased sarcolemma excitability (during a maximal contraction). It has been proposed that hypo-hydration could lead to a reduction in NM function (i.e., maximal strength), but compensatory mechanisms (i.e., reduced corticospinal inhibition) might occur to minimise force loss during a maximal voluntary contraction (MVC [Uddin et al. 2022a]), but this hypothesis is yet to be tested. In addition, Barley et al. (2018b) reported a reduction in high-intensity efforts 3 h and 24 h after 5% body mass reduction despite ad libitum fluid and food intake, indicating the need for understanding a time-course of recovery, and associated mechanisms, after dehydration and subsequent rehydration. Indeed, weight-classification sports vary in recovery times (e.g., judo *vs.* professional boxing), requiring athletes to compete as quickly as 2 h or as far as 36 h after a weigh-in. Therefore, it is crucial to investigate if a) the proposed compensatory mechanisms of reduced NM function are observed after dehydration of greater severities (e.g., 5% body mass reduction) and b) what the recovery time-course of such changes/compensations are after fluid restoration.

While TMS and MNS provide an indirect assessment of neural transmission efficacy between the brain and skeletal muscle, blood-based biomarkers of the CNS may provide a mechanistic reasoning for the observed changes in neural output. The blood–brain-barrier (BBB) plays a vital role in the tight regulation of exchange of ions, molecules and cells between the CNS and peripheral circulation (Daneman and Prat. 2015; Watson et al. 2006). Watson et al. (2006) reported that plain-water ingestion equal to the volume lost during exercise, attenuated the increase of Calcium Protein Binding b (S100b) in the peripheral bloodstream, indicating that rehydration might limit the increase in exercise-induced BBB permeability, and the associated dehydration process. It is possible that the osmotic pressure caused by hyperosmolality of the extracellular fluid causes a shift in fluids from the brain to the peripheral bloodstream (Cserr et al. 1987a, 1987b; De Boer and Breimer 1998; Kapural et al. 2002), and the shrinkage of the endothelial cells of the BBB, leading to transient opening of the tight junctions (Rapoport 2000). Unlike S100b, Ubiquitin C-terminal hydrolase -L1 (UCH-L1) is highly and specifically expressed in neurons and is pivotal in the removal of misfolded or oxidised proteins (Gong and Leznik 2007). To date, it is unknown if severe hypo-hydration, or variation in the dehydration and rehydration time-course, affects circulating peripheral UCHL-1, which would provide evidence of increased BBB permeability, as well as reduced neuronal integrity. Such a reduction may be synonymous with decreased NM function, or the proposed compensatory changes observed with TMS and MNS after hypo-hydration (Uddin et al. 2022a). Furthermore, if blood biomarkers related to BBB integrity are affected by hypohydration, this is likely to have important clinical implications, since their contribution to the identification of traumatic brain injury would be more questionable if fluid imbalance is likely to have occurred in close proximity to the measurement (Papa 2016).

The primary aims of the study were to investigate; (a) the corticospinal and peripheral responses to heat-induced hypo-hydration (intracellular dehydration) and (b) the time-course of NM function resulting from gradual and rapid rehydration. A secondary aim of the study was to investigate if UCH-L1 (a systemic blood marker linked to BBB and neuronal integrity) concentration is affected by intracellular dehydration and subsequent rehydration strategies. We hypothesised that after intracellular dehydration, there would be a reduction in maximal strength, muscle contractility and corticospinal inhibition, increase in sarcolemma excitability and UCH-L1 concentrations. Therefore, we hypothesised a reduction in MVC, muscle twitch parameters and an increase in the TMS-evoked silent period and compound muscle action potential, after intracellular dehydration.

## Methods

### Ethical approval

Ethical approval was provided by the institutional ethics committee of St Mary’s University College (SMEC_2018-19_055) which was conducted in accordance with the 2013 Helsinki declaration, besides pre-registration on an open database. All participants provided written informed consent before data collection.

### Participants

A total of 12 healthy male participants (29 ± 7 yr; 86.4 ± 9.5 kg; 185 ± 12 cm) were assigned (via a randomised crossover design) to undergo a control or experimental protocol, intended to induce a body mass (BM) reduction of ~ 5%. Before commencement, informed consent, a medical screening, and TMS screening questionnaire (Rossi et al. 2021) for any pre-existing conditions were obtained. Participants were required to be physically active, free from injury (6 months), not taken part in outdoor hot-weather training (3 months) and have no pre-existing medical conditions. All exercise trials were separated by a minimum of 7 days to minimise tiredness from the previous trial. Participants were requested to keep a personal food diary from the night before the trial, then replicate this for the subsequent trials. It was also requested that they avoid strenuous exercise, and the consumption of nutritional supplementation (e.g., caffeine) and alcohol.

### Experimental design

Participants took part in a randomised crossover study, with a control condition (CON), heat-induced dehydration with rapid rehydration (RHY2) and heat-induced dehydration with gradual rehydration (RHY24) condition. The control trial was the completion of a prolonged submaximal cycling (Monark, Varburg, Sweden) protocol (3 h at 60 W) in temperate conditions (25 ± 5 °C, 30 ± 3% relative humidity), whereby electrolyte fluid intake was permitted throughout, to account for body mass reduction every 30 min. Participants undergoing both dehydration trials completed the same exercise protocol twice, but in an environmental chamber (Sporting Edge, UK) under a hotter dry-bulb temperature of 45 ± 3 °C and relative humidity of 35 ± 3%. During this time, participants were restricted from fluid intake, to induce a 5% reduction of body mass through dehydration (Barley et al. 2018b).

Pre-testing dietary intake was controlled in the form of a standardised breakfast meal, consisting of rice cereal, milk, bread, butter, jam, and orange juice (Tesco, Welwyn Garden City, Hertfordshire, UK) and water (10 mL/kg), providing 20% of their estimated energy requirements (EER) and 1 g of carbohydrate/kg body mass (Evans et al. 2017a, b). Post-testing standardised meals consisted of a pasta or sandwich with a protein source, crisps and biscuits (Tesco, Welwyn Garden City, Hertfordshire, UK). All post-testing meals were homogenous, providing energy from 35 to 40% of their EER, and 14, 33 and 53% from proteins, fats and carbohydrates, respectively. All meal selections were provided to the participants and replicated for each trial. A text message was sent to each participant to reiterate the eating and drinking protocol after the trial. In addition, communication was maintained with each participant to ensure that there was no confusion, and the required fluid volume and meal selections were consumed at the correct times. No deviations for the meals consumed outside of the laboratory (i.e., breakfast and dinner) were reported.

Before each protocol, a post-breakfast urine sample, nude body mass, NM function, capillary and venous blood samples were obtained. Participants who were identified to be in a mildly dehydrated state (> 600 mOsml/kgH_2_O) prior to the protocol, were rehydrated with 250 mL of water followed by 30 min of seated rest before being re-tested and allowed to begin the protocol. Prior to commencement of the protocol, participants inserted a rectal thermometer (Edale Instruments Ltd., Cambridge, UK) 10 cm beyond their anal sphincter for assessment of core temperature. In addition, baseline resting blood pressure, heart rate, perceived thirst (Millard-Stafford et al. 2012) and thermal comfort (Bedford 1936) were assessed.

A cycling power output of 60 W was chosen to increase core temperature and promote sweating but minimise muscle glycogen depletion (Smith et al. 2000; Barley et al. 2018b). Upon completion, participants rested for 60 min in a thermo-neutral environment (19 ± 3 °C and  ~ 30% relative humidity), thereby facilitating core temperature to return to baseline. Throughout each protocol (every 30 min), participants were assessed for nude body mass (after being toweled down), whole body sweat rate (WBSR), rectal core temperature, blood pressure, heart rate and ratings of thermal comfort, perceived exertion, and thirst. In the case of (1) heart rate exceeding 180 beats/min for five consecutive minutes, (2) rectal temperature exceeding 39.5 °C, (3) displaying signs or symptoms of an exercise-induced heat illness (Coris 2004), or (4) a request to stop exercising, the protocol ceased, and participants were placed in a thermo-neutral environment to allow cooling and recovery (Judelson et al. 2007). In the case of participants dehydrating too rapidly (i.e., reaching near to 5% dehydration target ahead of schedule) (> 1.75 L/h), participants consumed a small volume of water (250 ml) during exercise, to ensure only 5% dehydration. Participants who were not predicted to reach a target loss of 5% body mass within the 3 h time frame (whole body sweat rate of < 1.25 L/h) were instructed to wear an additional layer of clothing (Track suit, Everlast, UK; Sweat suit, Everlast, UK). Participants were permitted to urinate post-protocol, following a body mass measurement, to offset fluid losses unrelated to heat-stress.

### Rehydration protocol

**Fig. 1:**
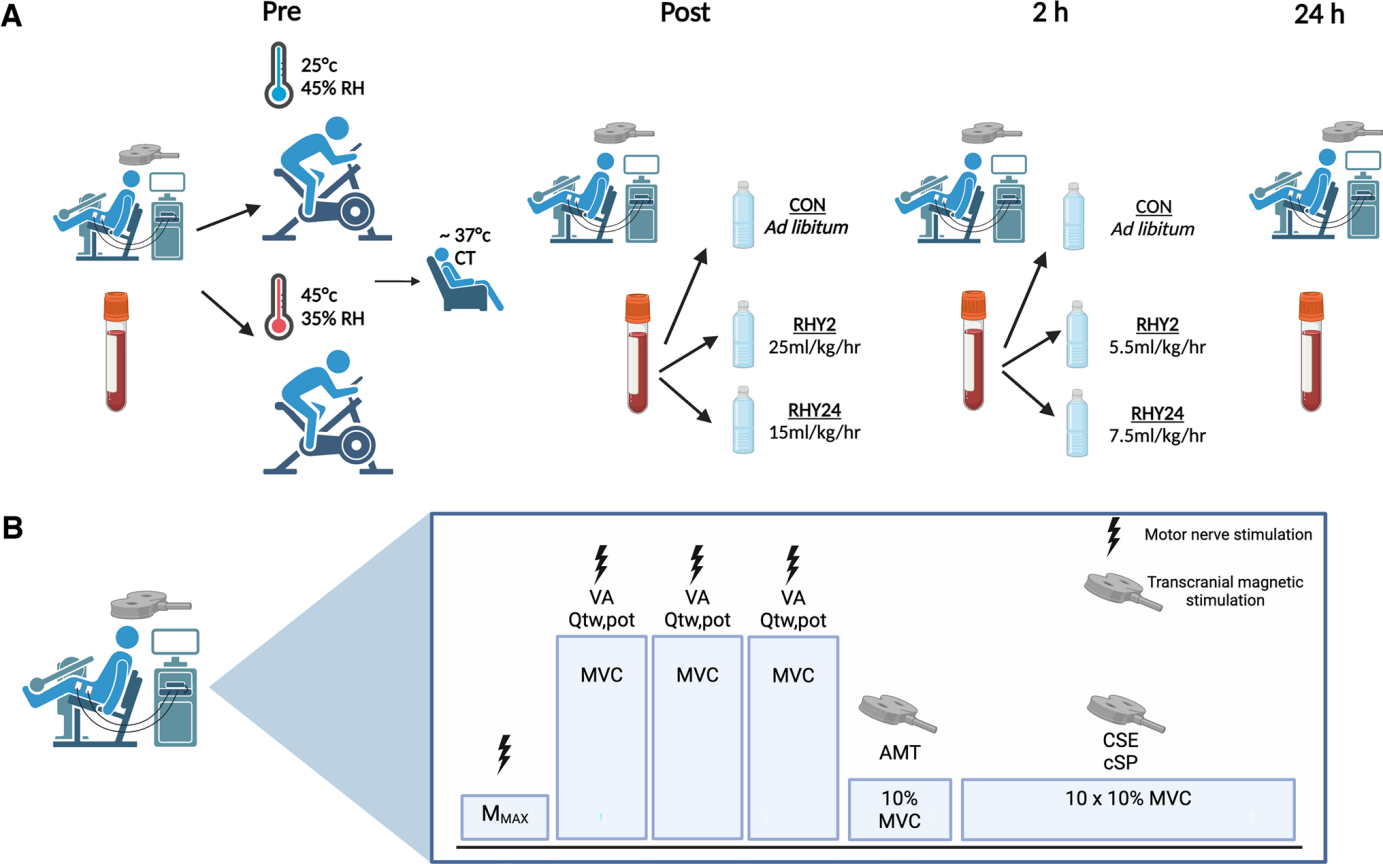
**A** Experimental design for all three conditions and, **B** breakdown of transcranial and peripheral motor nerve stimulation protocol. *AMT* active motor threshold, *cSP* cortical silent period, *CT* core temperature, *MEP* motor evoked potential, *M*_*MAX*_ compound muscle action potential, *MVC* maximum voluntary contraction, *Qtw,pot* potentiated twitch, *RH* relative humidity, *VA* voluntary activation

During the control trial, participants were permitted to consume an electrolyte beverage throughout the protocol, which was calculated as: 1.5 × body mass reduction (as observed in the 30 min interval body-mass recordings). Participants undergoing rapid rehydration (RHY2) aimed to achieve complete rehydration in 120 min through the consumption of an electrolyte beverage (25 mL/kg/h) in tapered stages. Thereafter, they consumed 5.5 mL/kg/h for the remaining 8 h. The second experimental trial rehydrated over a 24 h period (RHY24) and were instructed to consume 15 mL/kg/h of the electrolyte beverage in tapered stages in the first 120 min, followed by 7.5 mL/kg/h for the remaining hours. Consumption of fluids began 60 min after completion of the dehydration protocol to allow for cooling and assessment of NM function, except the control group, who were able to drink ad libitum. Rehydration electrolyte beverages were consumed in the form of water (obtained from a cool-water dispenser) and a commercial rehydration tablet, consisting of 2% CHO, 30 mmol/L sodium, 12.34 mmol/L potassium and 24.68 mmol/L chloride (Oral Rehydration Solutions, Clinova Ltd., Southampton, UK). Assessment of fluid, electrolyte and dietary intake throughout the post-testing period was achieved through measurement of urine volume, plasma volume, electrolytes and the provided dietary choices. A summary of the protocol can be found in Fig. 1.

### Neuromuscular function

#### Maximal voluntary force

A calibrated load cell was used to measure knee extensor force (N) during voluntary and evoked contractions. The load cell was fixed to a custom-built chair and connected to a noncompliant cuff attached around the participant’s right leg, superior to the malleoli. Participants sat upright in the chair with the hips and knees at 90° of flexion and were instructed to grasp the handles on the side of the chair for support during contractions. To quantify maximal force, participants were instructed to fold their arms across their chests and avoid upper body movement, hip flexor contractions, and ankle dorsiflexion. They were given verbal encouragement and visual feedback from a computer monitor to help them achieve a maximal voluntary contraction (MVC). Each attempt lasted for a duration of 3 s and was followed by a 1 min rest period. The highest sustained plateau during these attempts was recorded as the MVC. Each attempt had a 3 s duration and was separated by 1 min; the highest sustained plateau of which was recorded as the MVC. Electromyographical (EMG) activity was recorded from the rectus femoris (RF), and biceps femoris (BF) in accordance with SENIAM guidelines. Thorough skin preparation was ensured through shaving hair, removal of dead epithelial cells and wiping with isopropyl alcohol swabs. Surface electrodes (Philips, UK) were then placed 2 cm apart over the muscle bellies and a reference electrode placed over the patella. Electrode placement was marked with indelible ink to ensure a consistent placement post-trial and between trials.

Signals were amplified: gain × 1000 for EMG and × 300 for force (CED 1902, Cambridge Electronic Design, Cambridge, UK), band-pass filtered (EMG only: 20–2000 Hz), digitised (4 kHz; CED 1401, Cambridge Electronic Design), acquired and analyzed off-line (Signal v6.06, Cambridge Electronic Design).

#### Motor nerve stimulation

Single, electrical stimuli (200 μs pulse width) was delivered to the right femoral nerve through surface electrodes (CF3200, Nidd Valley Medical Ltd, North Yorkshire, UK) using a constant-current stimulator (DS7AH, Digitimer Ltd, Welwyn Garden City, Hertfordshire, UK). In line with previous literature (Sidhu et al. 2009a, b; Goodall et al. 2012), the cathode was positioned over the nerve, high in the femoral triangle, while the anode was placed midway between the greater trochanter and the iliac crest. Single electrical stimuli were delivered to the relaxed muscle beginning at 40 mA and increased by 20 mA until a plateau occurred in twitch amplitude and compound muscle action potential (M_MAX_) (Deely et al. 2022). Supramaximal stimulation was delivered by increasing the final stimulator output intensity by a further 30%. The positions of the stimulating electrodes were also marked with indelible ink to ensure consistent placement. Voluntary activation (VA) was assessed using the twitch interpolation method (Merton 1954) and quantified by comparing the amplitude of the superimposed twitch force (SIT) to the potentiated twitch (Qtw,pot) that was delivered 3 s after each MVC. The following equation was used offline: VA (%) = [1 – (SIT/(Qtw,pot)) × 100].

#### Transcranial magnetic stimulation

Single-pulse TMS was delivered using a magnetic stimulator (Magstim BiStim^2^, The Magstim Company Ltd, Whitland, UK) with a double cone coil (D110 The Magstim Company Ltd, Whitland, UK). Hotspot identification required low-level tonic contractions (10% MVC) of the rectus femoris and began with five pulses delivered over the vertex (measured as the intersection between 50% of the distance between the inion and nasion, and 50% between each tragus). An additional five pulses were then delivered at different locations along M1, with the mean motor evoked potential (MEP) amplitude value from the five pulses at each location calculated. The location over M1 with the highest mean rectus femoris MEP peak-to-peak amplitude, was designated as the motor hotspot and marked with a semi-permanent marker. To establish the TMS intensity, an isometric contraction of the quadriceps (10% MVC) was used to stabilise the contraction, while active motor threshold (AMT) was determined as the lowest stimulation intensity required to evoke peak-to-peak MEP greater than 200 μV in five out of 10 consecutive pulses (Rossini et al. 1994). The AMT was recorded as a percentage of maximal stimulator output (33 ± 7%). All remaining pulses were subsequently delivered at 130% of AMT during all the experimental procedures, with a total of 10–15 pulses at each time-point and a rest period of 15 s between each stimulus (Pagan et al. 2023). Visual feedback was provided on a monitor (1 m from the participant), with a horizontal line to represent % MVC.

Peak-to-peak amplitudes of the evoked MEPs and M_MAX_ were measured offline observing amplified EMG responses (D440 2, Digitimer North America LLC, USA). MEP amplitudes from single-pulse stimuli were averaged from 10 pulses (MEP_RAW_) and were subsequently normalised to M_MAX_ amplitude using the following equation: MEP/M_MAX_ = [(MEP/M_MAX_) × 100]. Duration of the TMS-evoked cortical silent period (cSP) was assessed from the stimulus artefact to the resumption of background EMG via visual inspection (Damron et al. 2008).

### Blood measures

Vacutainers (6 mg) containing lithium heparin and serum separating gel (SST) were used to collect venous blood samples, which were centrifuged either immediately (plasma) or after 30 min (serum), for 15 min at 1000 g at 4 °C (Mikro 220 R Hettich, Tuttlingen, Germany). Plasma and serum supernatant were aliquoted to microcentrifuge tubes and stored at − 80 °C until analysis. Serum Ubiquitin Carboxyl-terminal Hydrolase isoenzyme L1 (UCH-L1) concentration was determined using a commercially available enzyme immunoassay kit (abx251226, Abbexa Ltd, Cambridge, UK) per the manufacturer’s instructions. The detection range was 100–2300 pg/ml with a sensitivity of 47 pg/mL, with inter- and intra-assay coefficients of variation below < 10%. All assays were run in duplicate. To calculate PV change and correct concentrations for any changes in PV with dehydration, a capillary blood sample was taken pre- and post-exercise and hematocrit was measured using a micro-hematocrit centrifuge and reader (Hawksley, Sussex, UK). All blood samples were obtained after 30 min rest in a semi-reclined position, with arm height constant. Percent change in plasma volume (PV) was calculated using the following formula: % change in PV = (100/[100 Hct pre]) × 100([Hct pre − Hct post]/Hct post) (Dill and Costill 1974; Van Beaumont 1972).

### Urine measures

For the assessment of baseline hydration status (urine osmolality) and fluid retention, participants were instructed to collect midstream samples (50 ml) of urine 2 h post-breakfast and all urine (24 h urine volume) post-dehydration. An Osmocheck refractometer (Vitech Scientific Ltd, West Sussex, UK) was used to provide an indicative reading of urine osmolality (U_osm_) with a manufacturer’s reported testing accuracy of ± 20 mOsml/kgH^2^O and between-run coefficient (CV) of 0.3%. Participants were also requested to report fluid intake pre and post-trial completion.

### Statistical analysis

All data were assessed for normality and sphericity using a visual inspection of the Q-Q plots and Mauchly’s test of sphericity, respectively. Where data were parametric, the dependent variables were assessed using a two-way (Condition [CON, RHY2, RHY24] x Time [PRE, POST, POST2, POST24] repeated measures analysis of variance (ANOVA). Mid-protocol variables were similarly analyzed, but with a difference in time points (Condition [CON, RHY2, RHY24] x Time [Baseline, 30, 60, 90, 120, 150, 180, Rest]). Follow-up Bonferroni post hoc analyses were conducted if a significant main and interaction effect was observed. Data are presented as mean ± SD, and all statistical analyses were carried out using SPSS for Macintosh v28 (SPSS Inc., USA), with a significance level of *p* ≤ 0.05 applied. Effect sizes are reported as partial eta squared (η_p_^2^) with a threshold of 0.01, 0.06 and 0.14 as a small, medium and large effect, respectively (Cohen 1973).

## Results

### Neuromuscular function

**Fig. 2:**
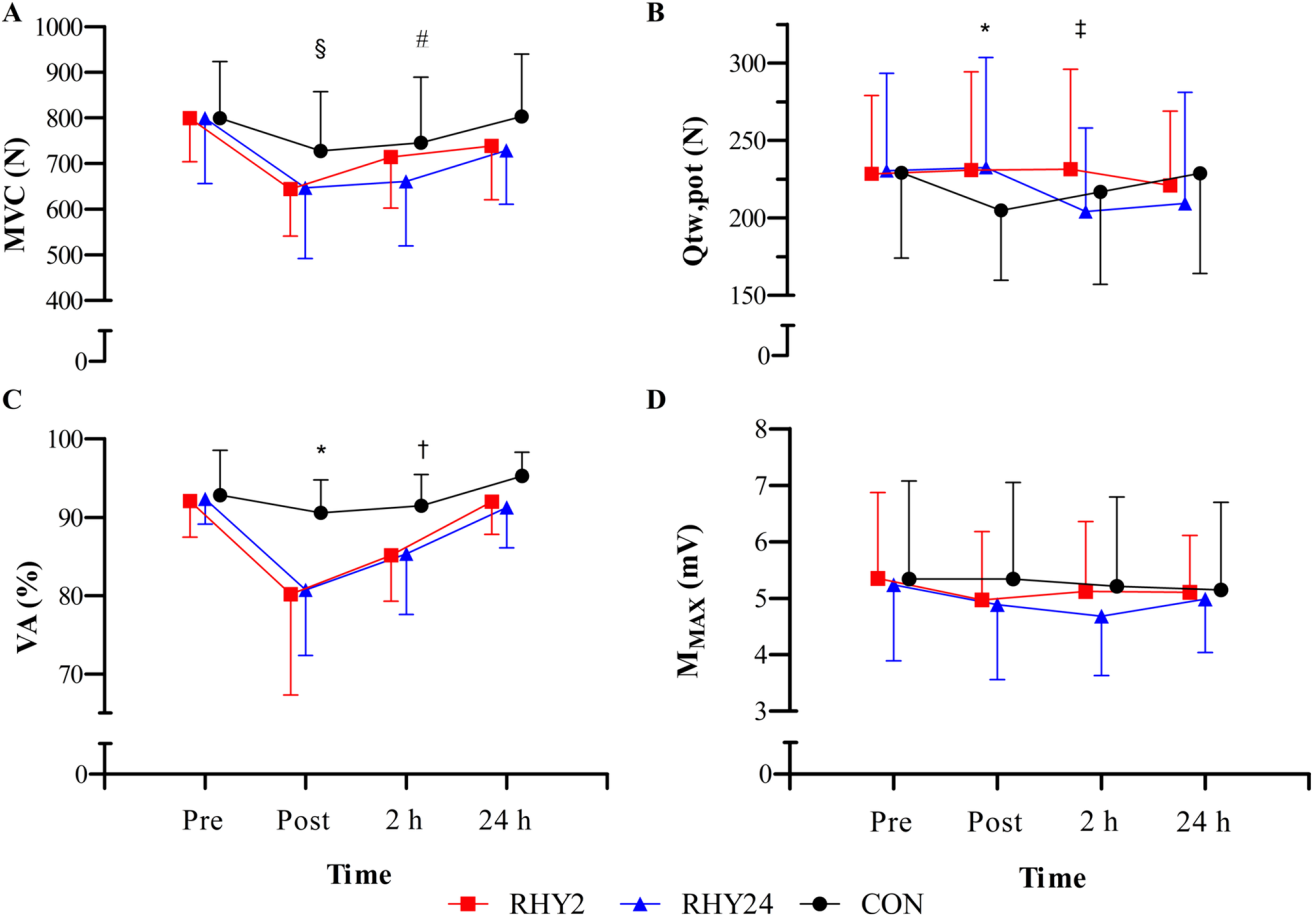
MVC (**A**), Qtw,pot (**B**), VA (**C**), and M_MAX_ (**D**) across time-points for each condition. Main interaction effects: Between condition differences for: * CON *vs.* RHY2, † CON *vs.* RHY24, ‡ RHY2 *vs.* RHY24. Within condition difference for: § RHY2 and RHY24 only, # RHY2 only. Time points are jittered for visual clarity

MVC reduced significantly across time (F_(3,33)_ = 34.9, *p* < 0.001, η_p_^2^ = 0.76) and a condition*time interaction (F_(6,66)_ = 2.3, *p* = 0.044, η_p_^2^ = 0.17) was observed for MVC. Post-hoc analyses determined no significant changes in the control group across time points; however, MVC significantly reduced between PRE and POST in the RHY2 condition (−19.3%, *p* < 0.001) and RHY24 condition (−19.1%, *p* = 0.001). There was also a significant improvement of MVC between POST and POST2 (8.7%, *p* = 0.004) in the RHY2 condition only (Fig. 2).

A condition*time interaction effect was observed for Qtw,pot (F_(6,66)_ = 4.1, *p* = 0.002, η_p_^2^ = 0.27), with post-hoc analyses demonstrating a significant reduction in Qtw,pot at POST during CON relative to the RHY2 condition (−11.5%, *p* = 0.045), and at POST2 during RHY24 relative to the RHY2 condition (−10.5%, *p* = 0.015).

A main effect of condition (F_(2,22)_ = 6.6, *p* = 0.006, η_p_^2^ = 0.37), time (F_(3,33)_ = 26.183, *p* < 0.001, η_p_^2^ = 0.70) and condition*time interaction effect (F_(2.548, 28.029)_ = 3.3, *p* = 0.043, η_p_^2^ = 0.23) were observed for VA. Post-hoc analyses showed VA at POST was lower during RHY24 *vs.* CON (−10.5%, *p* = 0.037), and at POST2, lower during RHY2 *vs.* CON (−6.3%, *p* = 0.017). No main effects (*p* = 0.407) or interactions (*p* = 0.593) were observed for M_MAX._

### Corticospinal measures

**Fig. 3:**
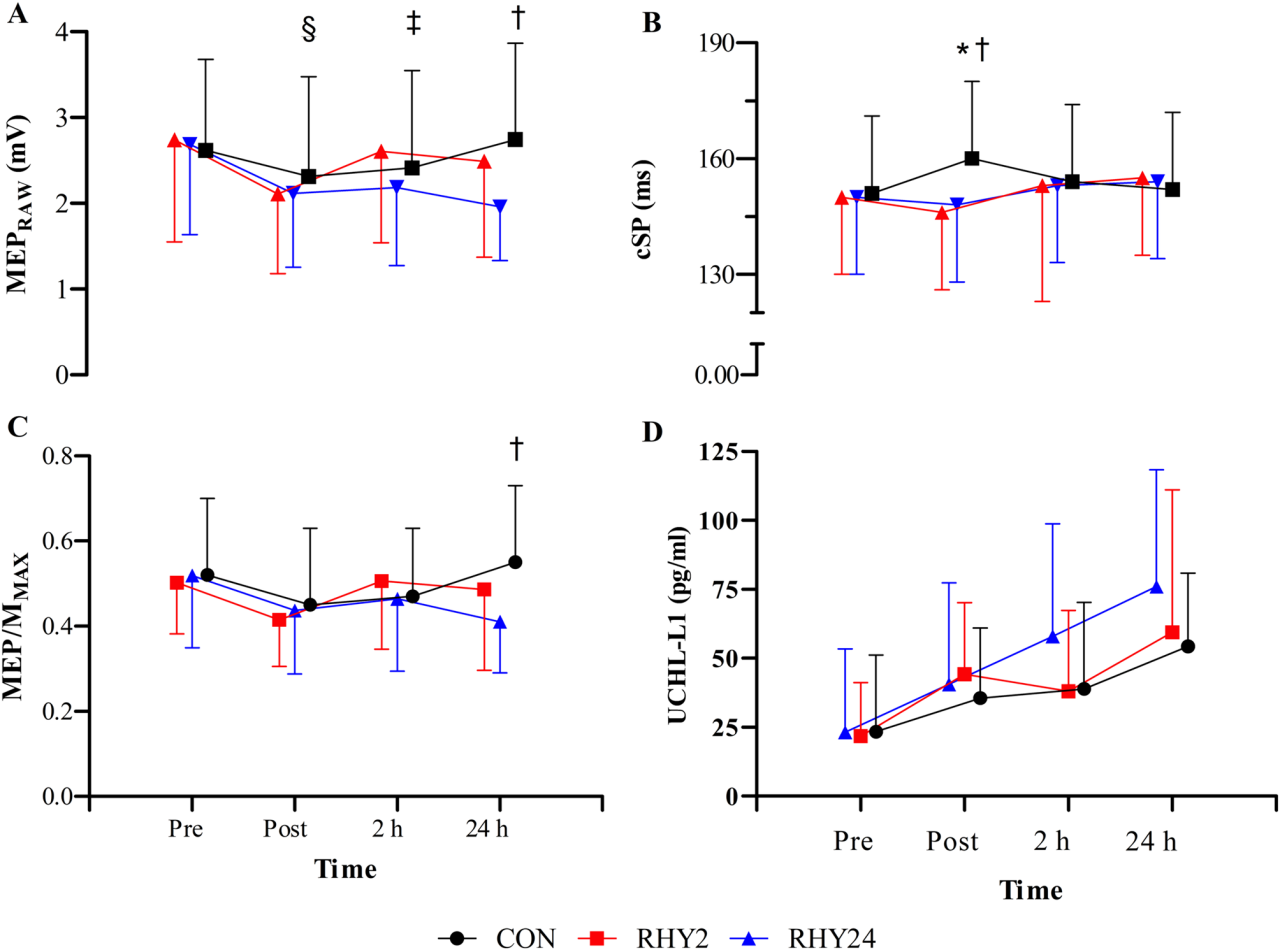
MEP_RAW_ (**A**), cSP (**B**), MEP/M_MAX_ ratio (**C**), and UCH-L1 (**D**; *n* = 10) across time-points for each condition. Main interaction effects: Between condition differences for: * CON *vs.* RHY2, † CON *vs.* RHY24, ‡ RHY2 *vs.* RHY24. Within condition difference for: § RHY2 and RHY24 only. Time points are jittered for visual clarity

There was a main effect of time (F_(3,33)_ = 7.3, *p* < 0.001, η_p_^2^ = 0.40) and interaction effect (F_(6,66)_ = 2.8, *p* = 0.018, η_p_^2^ = 0.20) for MEP_RAW_. Post-hoc analyses demonstrated a higher MEP amplitude at POST2 during RHY2, relative to the RHY24 condition (8.9%, *p* = 0.02), and a lower MEP amplitude at POST24 during RHY24, relative to the CON condition (−10.3%, *p* = 0.018) (Fig. 3A). In addition, there were within-condition differences at PRE *vs.* POST for RHY2 (−20.9%, *p* = 0.025) and RHY24 (−20.2%, *p* = 0.045) (Fig. 3A). No condition effect was observed (*p* = 0.166).

An interaction effect was observed for cSP (F_(6,66)_ = 5.4, *p* < 0.001, η_p_^2^ = 0.33). Post-hoc analyses showed a significant lengthening in POST exercise cSP duration during the CON condition (0.16 ± 0.006 ms [6.3%]) relative to the RHY2 condition (0.146 ± 0.005 ms, *p* = 0.011) and RHY24 condition (0.148 ± 0.005 ms, *p* = 0.002) (Fig. 3B).

A main effect of time (F_(3,33)_ = 4.6, *p* = 0.09, η_p_^2^ = 0.29) and interaction effect (F_(6,66)_ = 2.8, *p* = 0.016, η_p_^2^ = 0.21) was observed for MEP/M_MAX_ ratio. Post-hoc analyses showed a significant decrease from PRE (0.51 ± 0.04) to POST (0.43 ± 0.03) MEP/M_MAX_ ratio (*p* = 0.009) across all conditions. Additionally, MEP/M_MAX_ ratio at POST24, was significantly lower during RHY24, relative to the CON condition (−25.6%, *p* = 0.026). No main (*p* = 0.196) or interaction effects (*p* = 0.946) were observed for AMT.

### Serum UCH-L1

A time effect was observed for UCH-L1 (F_(3,27)_ = 9.3, *p* < 0.001, η_p_^2^ = 0.51). Post-hoc analyses showed a significant increase in peripheral UCH-L1 concentration at all time points (POST [*p* = 0.015]; POST2 [*p* = 0.022]; POST24 [*p* = 0.009]) relative to baseline (*n* = 10; Fig. 3D). No condition effect was observed (*p* = 0.816).

### Body mass, plasma volume and fluid intake

Body mass was significantly higher in the CON condition (F_(2,22)_ = 20.1, *p* < 0.001, η_p_^2^ = 0.65), and at baseline relative to all time points (F_(3,33)_ = 261.6, *p* < 0.001, η_p_^2^ = 0.96). An interaction effect between the dehydration conditions and CON was also observed (F_(2.5,27.9)_ = 140.9, *p* < 0.001, η_p_^2^ = 0.93) for body mass (Table 1). A main effect of time (F _(2,22)_ = 243.6, *p* < 0.001, η_p_^2^ = 0.96) and an interaction effect were also observed in delta PV change (F _(4,44)_ = 18.3, *p* > 0.001, η_p_^2^ = 0.63). Pairwise differences are reported in Table 1.

**Table 1:** Body mass and plasma volume change across time points

	PRE	POST	POST2	POST24
Body mass (kg)				
CON	86.6 ± 9.4	86.4 ± 9.5	86.9 ± 9.6	86.7 ± 9.5
RHY2	86.5 ± 9.5	82.5 ± 9.1†	86.6 ± 9.3‡	86.6 ± 9.1
RHY24	86.4 ± 9.3	82.4 ± 8.9†	84.8 ± 9.0*	86.5 ± 9.3
ΔPV (%)				
CON	0.0	−3.4 ± 2.0*	1.5 ± 2.8	3.9 ± 4.5
RHY2	0.0	−13.3 ± 2.3†	0.9 ± 3.6‡	3.9 ± 3.4
RHY24	0.0	−13.3 ± 3.6†	−5.6 ± 3.0*	1.6 ± 4.4

Values are means (± SD). ΔPV % change in plasma volume from baseline

*Significant pairwise difference from RHY2 and RHY24

†Significant pairwise difference from CON

‡Significant pairwise difference from RHY24

### Mid-protocol variables

There were main and condition*time interaction effects for rectal temperature, heart rate, thermal comfort and thirst scale scores, with (*p* < 0.001). Post-hoc analyses showed significant differences between CON and both dehydration conditions (*p* < 0.005), but not between RHY2 and RHY24 (*p* > 0.005) (Table 2). Average fluid intake during RHY2 and RHY24 were 375.4 ± 68.0 mL/h and 330.8 ± 47.0 mL/h respectively. Urine volume during RHY2 and RHY24 was 168.2 ± 50.0 mL/h and 113.2 ± 26.2 mL/h, respectively. Consequently, fluid retention was 55.2 and 65.8% during RHY2 and RHY24, respectively.

**Table 2:** Mid-protocol variable change across trial duration

Time (min)		Baseline	30	60	90	120	150	180	Rest
Rectal temperature (°C)	CON	37.0 ± 0.1	37.3 ± 0.1*	37.5 ± 0.2*	37.6 ± 0.2*	37.6 ± 0.3*	37.6 ± 0.3*	37.5 ± 0.4*	37.2 ± 0.3
	RHY2	37.1 ± 0.2	37.6 ± 0.2	37.8 ± 0.2	38.1 ± 0.3	38.5 ± 0.3	38.7 ± 0.2	38.9 ± 0.3	37.3 ± 0.2
	RHY24	37.1 ± 0.2	37.5 ± 0.2	37.9 ± 0.2	38.2 ± 0.3	38.5 ± 0.3	38.8 ± 0.2	39.0 ± 0.2	37.2 ± 0.2
Heart rate (beats/min)	CON	74 ± 6	102 ± 7*	120 ± 8*	128 ± 5*	138 ± 6*	146 ± 8*	150 ± 5*	75 ± 5
	RHY2	76 ± 12	119 ± 10	137 ± 17	145 ± 18	157 ± 14	160 ± 12	162 ± 15	78 ± 4
	RHY24	78 ± 8	119 ± 11	136 ± 12	141 ± 13	152 ± 14	156 ± 10	164 ± 13	80 ± 13
Thermal comfort (-3 to + 3)	CON	0 ± 1	0 ± 0*	0 ± 1*	1 ± 1*	1 ± 0*	1 ± 1*	1 ± 1*	0 ± 1*
	RHY2	0 ± 1	1 ± 1	1 ± 1	2 ± 1	2 ± 1	3 ± 1	3 ± 0	0 ± 0
	RHY24	0 ± 0	0 ± 1	1 ± 1	1 ± 1	2 ± 1	2 ± 1	3 ± 1	0 ± 0
Thirst scale (1 to 7)	CON	2 ± 1	2 ± 1	2 ± 1*	3 ± 1*	2 ± 1*	2 ± 1*	2 ± 1*	2 ± 1*
	RHY2	2 ± 1	3 ± 2	4 ± 1	5 ± 2	6 ± 1	7 ± 1	7 ± 1	7 ± 0
	RHY24	2 ± 1	2 ± 1	4 ± 1	5 ± 1	6 ± 1	6 ± 1	7 ± 0	7 ± 0

Values are means (± SD)

*Significant pairwise difference from RHY2 and RHY24

## Discussion

The aims of the study were to (a) investigate the corticospinal and peripheral responses to low-intensity exercise, heat-induced hypo-hydration (intracellular dehydration) and rehydration and (b) the time-course of NM function resulting from gradual and rapid rehydration. Our findings indicate that NM function is impaired after heat-induced hypo-hydration, as characterised by a reduction in MVC of the lower-limb, but the mechanisms of impairment appear to differ from that of prolonged low-intensity exercise. Heat-induced hypohydration led to a reduction in voluntary activation and corticospinal excitability in both experimental conditions, while exercise while hydrated (CON) increased corticospinal inhibition and reduced muscle contractility. In addition, rapid rehydration partially restored maximal strength and corticospinal excitability after 2 h (RHY2), but corticospinal excitability remained depressed at 24 h despite NM function returning to baseline (RHY24).

Our findings add to a large body of evidence of MVC reduction due to hypo-hydration (Bosco et al. 1968; Torranin et al. 1979; Webster et al. 1990; Judelsen et al. 2007; Hayes and Morse 2010; Schofstall et al. 2001; Bigard et al. 2001; Bowtell et al. 2013; Reece et al. 2023). However, it is of interest that MVC increased from POST to POST2 during RHY2, but not during RHY24, indicating a partial improvement in strength with rapid restoration of fluid balance, but not with gradual fluid restoration. This agrees with the findings of Schofstall et al. (2001), where a restoration of maximal strength after two hours of rest and rehydration (1.5% body mass loss) was reported, whilst also speculating that more time might be required to restore strength losses after dehydration of larger magnitude. While it is generally recommended that rehydration is more efficient when fluid consumption is progressively tapered (Jones et al. 2010), this pattern may not be optimal to the restoration of maximal strength, and in part, this can be explained by changes in the central and peripheral nervous systems. Voluntary activation did not differ as a function of the rehydration strategy, indicating this restoration of maximal strength was not related to a reduction in central fatigue (Kent-Braun and LeBlanc 1996). On the other hand, the reduction in the Qtw,pot occurring immediately after exercise in the euhydrated state, and at POST2 during RHY24 indicates a reduced myofibrillar Ca^2+^ sensitivity and/or force produced at a cross-bridge level. Indeed, Periard et al. (2014) reported an increased muscle relaxation rate and decreased relaxation time after hyperthermia, yet a centrally mediated rate of activation to overcome the faster relaxation rates. It is plausible that the increase of muscle temperature had a residual effect on muscle-contractile properties, initially protecting the muscle from the effects of reduced contractility. Indeed, increased muscle temperature is reported to accelerate the opening and closing of voltage-gated Na^+^ channels, leading to a more rapid onset depolarization and faster muscle fiber conduction velocity (Rutkove et al. 1997). It is possible that as core temperature returned to baseline, but plasma volume remained ~ 7% below baseline, muscle volume remained below baseline, and Ca^2+^ sensitivity and myosin phosphorylation decreased as dehydration ensued (Hackney et al. 2012). Overall, hypo-hydration leads to a loss of maximal strength capacity, and acute losses (2 h) were partially attenuated by rapid restoration of fluids.

In the current study, a 12% reduction of VA was observed after both dehydration trials, which differs to the findings of others (Periard et al. 2012; Bowtell et al. 2014; Barley et al. 2018a), where VA (from TMS and MNS) and CSE were reported to be unaffected by a 2–5% reduction of body mass via heat-induced hypo-hydration and fluid restriction. However, our findings agree with Del Coso et al (2008), where a 5% reduction in VA was observed after 2 h of moderate-intensity exercise in the heat (3.8% body mass reduction), demonstrating that higher levels of hypo-hydration might be sufficient to impair VA (Bowtell et al. 2013). In addition, Del Coso et al (2008) observed similar changes in VA when exercising in the heat with plain water ingestion, but minimal changes to VA when ingesting a 6% carbohydrate electrolyte solution, implying carbohydrate availability influences VA. This might explain why rehydration type did not influence VA recovery after exercise, as both groups consumed a standardised meal during the recovery period, but it is worth noting that 2 h of gradual or rapid rehydration and recovery (in addition to a cooling period) is not sufficient to restore VA after heat-induced hypo-hydration. It is plausible that the sustained central fatigue could be attributed to mechanisms of supraspinal origin and the result of increased hypothalamic temperature. Goodall et al. (2015) and Ross et al. (2011) suggested that an increase in central fatigue was related to cerebrovascular responses to heat. Hypo-hydration also notably increases perceptions of fatigue and pain (Moyen et al. 2015; Perry et al. 2016; Bear et al. 2016; Ogino et al. 2014). It is possible that conscious signals originating from both central and peripheral afferent pathways could mediate behavior, such that motivation to perform simple motor tasks is reduced to minimise discomfort (Cabanac 2006). While the exercise modality in this study was less intense than that of Del Coso et al (2008), participants did not undergo preliminary heat acclimation training and exercised for 60 min longer, possibly leading to increased pain and discomfort, and the observed larger reductions in VA (~ 10%). Overall, VA is reduced after heat-induced hypo-hydration and recovery is not influenced by gradual or rapid rehydration strategies.

A novel finding of this study was the differential corticospinal responses to prolonged light-intensity exercise and hydration status, which may explain the reduction in NM function. The M_MAX_, is evoked when all the motor axons of a target muscle are activated simultaneously. By normalizing the MEP) amplitude to the M_MAX_ (expressed as MEP/M_MAX_), we introduce a methodological advantage. This normalization allows us to consider the relative contribution of the corticospinal volley within the pool of activated motor neurons. However, as Bowtell et al (2013) have previously demonstrated that the M_MAX_ changes during maximal contractions after hypo-hydration, corticospinal excitability was examined as both MEP_RAW_ and MEP/M_MAX_ in this study_._ No changes in sarcolemma excitability were observed in the present study; however, MEP/M_MAX_ significantly decreased after light-intensity exercise, and further decreased at 24 h for the RHY24 condition (relative to CON; Fig. 3C). This was equally observed with MEP_RAW,_ with the addition of MEP_RAW_ at POST2 being significantly higher after rapid rehydration (RHY2), relative to RHY24. This indicates a reduced excitability of several depolarised pyramidal tract neurones (Nitsche and Paulus 2000; Ridding and Rothwell 1997) when in a hypo-hydrated state, which we have previously hypothesised (Uddin et al. 2022a). It is of interest that MVC and MEP amplitude did not improve at POST2 during RHY24, and this was accompanied by a marked reduction in Qtw,pot (Fig. 2D). Our findings demonstrate that reduced maximal strength after hypo-hydration is accompanied with a reduction in CSE and VA, and despite a restoration of maximal strength and VA, this reduction in CSE may persist after gradual rehydration.

While we did not measure intracortical changes, our findings are supported by Bowtell et al. (2013), where corticospinal inhibition was unchanged among hypo-hydrated subjects (despite a reduction in force), but cSP was lengthened in euhydrated subjects (Fig. 3B). The cSP is attributed to a combination of spinal inhibitory mechanisms (i.e., after-hyperpolarization and recurrent inhibition of the motor neuron pool), and intracortical mechanisms mediated by slower metabotropic GABA_B_ receptors (via the opening of K^+^ channels) in the M1, leading to the reduction of corticospinal drive. Overall, this would imply that following euhydration, there is a preferential increase in synaptic efficacy of GABA_B_ neurones which act to increase synaptic efficiency of pyramidal cells, thus inhibiting them. The mechanisms for these differential responses in corticospinal excitability and inhibition warrant further investigation.

Our findings also show an effect of time in the change of a blood marker linked to BBB permeability after low-intensity exercise. Pre-clinical studies have suggested that physical exertion may upregulate genetic expression of UCH-L1 e.g., increased striatal and hippocampal mRNA expression of UCH-L1 in rats undergoing aerobic exercise (Liu et al. 2018, 2019; Kirchner et al. 2008). These findings were speculated to be the result of exercise-induced signaling from mTOR or calcium/calmodulin dependent protein kinases (Liu et al. 2019). Bazarian et al (2023) reported an increase in UCH-L1 concentrations post-physical exertion in humans and reported exercise duration (but not brain white matter integrity) to have a linear correlation to increases in UCH-L1. Our findings demonstrate no between-group differences in UCH-L1 concentrations, despite the numerically faster rate of rise in the RHY24 condition. These results are contrary to the findings of Watson et al. (2001), which showed a restoration of s100B concentrations after rehydration. Further research is required to understand if hypo-hydration has an isolated effect on blood markers of BBB integrity. Overall, our data might have important implications for the use of blood-based biomarkers, such as UCH-L1, as on-field point-of-care tests, as physical exertion (and possibly hydration status) may confound the results and do not appear to be associated with NM function.

There are some potential limitations of the current study, which include a lack of strict standardization of the rehydration beverage temperature. Whilst fluids were dispensed from the same water cooler in the laboratory, the beverages will have inevitably changed temperature across the longer 24 h periods. We are currently uncertain whether the assumed increases in fluid temperature would affect the variables we have measured, and improved methods to control fluid temperature would help to remove this uncertainty in future research. Further, based on the current sample size, it was not feasible to statistically account for the variation in the methods used to induce ~ 5% body mass reduction among a small number of the participants (e.g., participants requiring additional layers of clothing or additional rest during the protocol). In addition, the inability to investigate intracortical activity via the use of paired-pulse stimulation measures (e.g., long and short interval intracortical inhibition, as well as the addition of a no-exercise trial) was a limitation of the current study. Future studies should consider adopting these measures to provide mechanistic insights into the TMS-evoked silent period (as observed) and other intracortical changes of the CNS. This would further clarify the exclusive role of intracellular dehydration after exercise and heat-induced hypo-hydration.

### Practical implications

There has been a well-documented global increase in climatic temperatures, increasing the frequency, intensity, and duration of heat waves (Stocker 2014). These events place greater demand on the thermoregulatory system and, thus, heat loss mechanisms, such as thermal sweating (Maughan and Shirreffs 2004). Thermal strain and secondary sweat responses are a common cause of hypohydration, particularly during periods of physical activity (Sawka et al. 2015), which is a known risk factor for heat-related illnesses (Nelson et al. 2011). Among athletes or those from physically demanding occupations, thermal strain can be exacerbated, increasing the likelihood of heat-induced hypo-hydration (i.e., intracellular dehydration; Sawka 1992). Among these sub-populations, rehydration guidelines comprise the use of electrolytes, carbohydrates and protein, as well as rate and volume of fluid intake, which are considered to mitigate some of the deleterious effects of heat-induced hypo-hydration (Evans et al. 2017; Burke et al. 2021), yet the potential implication of rapid and progressive rehydration has been somewhat overlooked in relation to NM function. Therefore, for both the general population and athletes who are likely to face periods of thermal strain and subsequent fluid losses, our findings demonstrate the likely time-course of changes in NM function and how rapidly rehydrating across 2 h may partially improve muscle force production, which would not be the case with more progressive strategies. This suggests that athletes who undergo rapid weight loss in close proximity to their sporting event (e.g., boxers, judokas and mixed martial artists), are likely to experience a decrement in performance, particularly when fluids are not rapidly restored. In addition, the clinical utility of UCH-L1 as a blood-based biomarker of traumatic brain injuries is well established (Bazarian et al. 2018; Korley et al. 2021). Situations in which a rapid and accurate point-of-care diagnosis is required, are critical in combat and contact sporting environments. However, with the observed increases in the concentration of UCH-L1 after exercise, and the concurrent occurrences of intracellular dehydration and risk of brain injury in contact sports (Uddin et al. 2022b), further research is warranted to establish if physical activity may present as a confounder in the clinical utility of point-of-care blood tests, particularly in sporting events.

## Conclusion

In summary, our findings provide novel evidence of the different neurophysiological responses to heat-induced hypo-hydration, demonstrating an increase in corticospinal inhibition after low-intensity exercise with fluid ingestion, but a decrease in corticospinal excitability after intracellular dehydration. In addition, maximal strength is impaired after heat-induced hypo-hydration, which is likely a result of reduced corticospinal excitability and voluntary activation, but this can be partially attenuated by rapid restoration of fluids.

## Electronic supplementary material

Below is the link to the electronic supplementary material.

Supplementary file1 (DOCX 17 KB)

## Data Availability

The supporting data for this manuscript are available upon reasonable request to the authors.

## Appendix

See Table 3. 

**Table 3:** Summary of neuromuscular function and TMS variables presented as Δ percentage change across time points

		POST	POST2	POST24
Δ MVC (%)	CON	–8.65 ± 10.5	–7.0 ± 7.8	0.6 ± 8.8
	RHY2	–19.3 ± 9.5	–10.6 ± 9.9	–7.6 ± 9.4
	RHY24	–19.1 ± 11.2	–16.6 ± 12.8	–8.1 ± 8.9
Δ VA (%)	CON	–2.1 ± 8.0	–1.1 ± 7.6	2.9 ± 6.6
	RHY2	–13.3 ± 10.9†	–7.4 ± 5.9	0.0 ± 5.0
	RHY24	–12.6 ± 7.8	–7.6 ± 7.3†	1.1 ± 5.6
Δ Qtw,pot (%)	CON	–9.9 ± 6.2‡	–5.2 ± 10.9	–0.5 ± 8.0
	RHY2	0.2 ± 12.4	0.1 ± 8.5	–2.9 ± 8.0
	RHY24	1.6 ± 18.0	–10.4 ± 11.5*	–10.0 ± 12.9
Δ MEP_RAW_ (%)	CON	–12.9 ± 13.8	–7.7 ± 16.7	7.0 ± 27.4
	RHY2	–20.9 ± 17.1	–6.7 ± 16.2‡	–7.2 ± 19.5
	RHY24	–20.2 ± 20.0	–15.6 ± 22.1	–17.3 ± 24.9†
Δ MEP/M_MAX_ (%)	CON	–13.0 ± 12.8	–6.7 ± 16.6	9.8 ± 25.1
	RHY2	–15.5 ± 19.7	0.1 ± 13.8	–4.1 ± 23.5
	RHY24	–14.2 ± 22.1	–10.0 ± 18.8	–15.8 ± 14.8†
Δ cSP (%)	CON	6.3 ± 4.0*‡	1.9 ± 4.5	1.0 ± 3.2
	RHY2	–2.1 ± 5.2	1.9 ± 7.9	3.5 ± 8.9
	RHY24	–0.6 ± 2.9	1.9 ± 2.8	3.1 ± 4.9

Values Δ % change from baseline (± SD)

*Significant pairwise difference from RHY2

† Significant pairwise difference from CON

‡ Significant pairwise difference from RHY24

## Abbreviations

AMT: Active motor threshold
BBB: Blood-brain-barrier
CNS: Central nervous system
CSE: Corticospinal excitability
cSP: Cortical silent period
EEG: Electroencephalography
EMG: Electromyography
GABA: γ-Aminobutyric acid
fMRI: Functional magnetic resonance imaging
HRT: Half-relaxation time
M_MAX_: Maximum motor unit potential
MNS: Motor nerve stimulation
MVC: Maximum voluntary contraction
NM: Neuromuscular
PNS: Peripheral nervous system
Qtw,pot: Potentiated twitch
RWL: Rapid weight loss
S100b: Calcium binding protein b
UCH-L1: Ubiquitin carboxyl-terminal hydrolase
VA: Voluntary activation
TMS: Transcranial magnetic stimulation
