# Supplementary material for: The effects of exercise, heat-induced hypo-hydration and rehydration on blood–brain-barrier permeability, corticospinal and peripheral excitability
Source: Eur J Appl Physiol. 2024 Sep 28;125(2):535–50. doi: 10.1007/s00421-024-05616-x (PMC11829906; doi:10.1007/s00421-024-05616-x)
Supplement: Supplementary file 1 — Supplementary file1 (DOCX 17 KB) [file 421_2024_5616_MOESM1_ESM.docx]

Appendix

**Table 1.** Summary of neuromuscular function and TMS variables presented as Δ percentage change across time points.

|  |  | **POST** | **POST2** | **POST24** |
| --- | --- | --- | --- | --- |
| **Δ MVC (%)** | **CON** | -8.65 ± 10.5 | -7.0 ± 7.8 | 0.6 ± 8.8 |
|  | **RHY2** | -19.3 ± 9.5 | -10.6 ± 9.9 | -7.6 ± 9.4 |
|  | **RHY24** | -19.1 ± 11.2 | -16.6 ± 12.8 | -8.1 ± 8.9 |
| **Δ VA (%)** | **CON** | -2.1 ± 8.0 | -1.1 ± 7.6 | 2.9 ± 6.6 |
|  | **RHY2** | -13.3 ± 10.9† | -7.4 ± 5.9 | 0.0 ± 5.0 |
|  | **RHY24** | -12.6 ± 7.8 | -7.6 ± 7.3† | 1.1 ± 5.6 |
| **Δ Qtw,pot (%)** | **CON** | -9.9 ± 6.2‡ | -5.2 ± 10.9 | -0.5 ± 8.0 |
|  | **RHY2** | 0.2 ± 12.4 | 0.1 ± 8.5 | -2.9 ± 8.0 |
|  | **RHY24** | 1.6 ± 18.0 | -10.4 ± 11.5* | -10.0 ± 12.9 |
| **Δ MEP_RAW_ (%)** | **CON** | -12.9 ± 13.8 | -7.7 ± 16.7 | 7.0 ± 27.4 |
|  | **RHY2** | -20.9 ± 17.1 | -6.7 ± 16.2‡ | -7.2 ± 19.5 |
|  | **RHY24** | -20.2 ± 20.0 | -15.6 ± 22.1 | -17.3 ± 24.9† |
| **Δ MEP/M_MAX_ (%)** | **CON** | -13.0 ± 12.8 | -6.7 ± 16.6 | 9.8 ± 25.1 |
|  | **RHY2** | -15.5 ± 19.7 | 0.1 ± 13.8 | -4.1 ± 23.5 |
|  | **RHY24** | -14.2 ± 22.1 | -10.0 ± 18.8 | -15.8 ± 14.8† |
| **Δ cSP (%)** | **CON** | 6.3 ± 4.0*‡ | 1.9 ± 4.5 | 1.0 ± 3.2 |
|  | **RHY2** | -2.1 ± 5.2 | 1.9 ± 7.9 | 3.5 ± 8.9 |
|  | **RHY24** | -0.6 ± 2.9 | 1.9 ± 2.8 | 3.1 ± 4.9 |

Values Δ % change from baseline (± SD). * Significant pairwise difference from RHY2. † Significant pairwise difference from CON. ‡ Significant pairwise difference from RHY24.
